# Gcn5 and Esa1 function as histone crotonyltransferases to regulate crotonylation-dependent transcription

**DOI:** 10.1074/jbc.RA119.010302

**Published:** 2019-11-07

**Authors:** Leonie Kollenstart, Anton J. L. de Groot, George M. C. Janssen, Xue Cheng, Kees Vreeken, Fabrizio Martino, Jacques Côté, Peter A. van Veelen, Haico van Attikum

**Affiliations:** ‡ Department of Human Genetics, Leiden University Medical Center, Einthovenweg 20, 2333 ZC, Leiden, The Netherlands; § Center for Proteomics and Metabolomics, Leiden University Medical Center, Albinusdreef 2, 2333 ZC, Leiden, The Netherlands; ¶ St. Patrick Research Group in Basic Oncology, Laval University Cancer Research Center, Centre de Recherche du Centre Hospitalier Universitaire de Québec-Axe Oncologie, Québec City, QC G1R 3S3, Canada; ‖ Centro de Investigaciones Biológicas (CIB), Consejo Superior de Investigaciones Científicas (Spanish National Research Council), (CSIC), Ramiro de Maeztu 9, 28040 Madrid, Spain

**Keywords:** epigenetics, histone modification, gene transcription, post-translational modification (PTM), chromatin modification, crotonylation, Esa1-Yng2-Epl1 (Piccolo NuA4) complex, Gcn5-Ada (ADA) complex

## Abstract

Histone post-translational modifications (PTMs) are critical for processes such as transcription. The more notable among these are the nonacetyl histone lysine acylation modifications such as crotonylation, butyrylation, and succinylation. However, the biological relevance of these PTMs is not fully understood because their regulation is largely unknown. Here, we set out to investigate whether the main histone acetyltransferases in budding yeast, Gcn5 and Esa1, possess crotonyltransferase activity. *In vitro* studies revealed that the Gcn5-Ada2-Ada3 (ADA) and Esa1-Yng2-Epl1 (Piccolo NuA4) histone acetyltransferase complexes have the capacity to crotonylate histones. Mass spectrometry analysis revealed that ADA and Piccolo NuA4 crotonylate lysines in the N-terminal tails of histone H3 and H4, respectively. Functionally, we show that crotonylation selectively affects gene transcription *in vivo* in a manner dependent on Gcn5 and Esa1. Thus, we identify the Gcn5- and Esa1-containing ADA and Piccolo NuA4 complexes as *bona fide* crotonyltransferases that promote crotonylation-dependent transcription.

## Introduction

Post-translational modifications (PTMs)^2^ on histones provide chromatin with a layer of regulation for vital processes such as gene transcription, DNA replication, and DNA damage repair (1–3). The maintenance of PTMs through modifying enzymes called “writer” and “eraser” proteins, which add and remove PTMs, respectively, is critical for these processes. The identification and characterization of writers and erasers has brought insight into the functional relevance of many PTMs, most notably that of histone acetylation, in gene transcription (4, 5). Through various MS-based approaches, the repertoire of PTMs has been expanding rapidly. The more notable among these are the nonacetyl histone lysine acylation modifications such as crotonylation, butyrylation, and succinylation (6–10).

Histone acylation marks, similar to those induced through acetylation, are linked to active gene transcription (7, 11, 12). In recent years, the first writers and erasers have been identified for various acylation marks. The well-characterized histone acetyltransferase (HAT) p300 and KAT2A (hGCN5) possess crotonyltransferase and succinyltransferase activity, respectively, toward histones (11, 13), whereas class I histone deacetylases (HDACs) (HDAC1, HDAC2, HDAC3, and HDAC8) and class III HDACs (SIRT1, SIRT2 and SIRT3) are capable of decrotonylating histones (14–16). However, compared with histone acetylation, the regulation of crotonylation, butyrylation, and succinylation is still poorly understood. This may be in part due to specificity issues of some antibodies, particularly those against histone crotonylation and butyrylation (17, 18).

For histone acetylation, the identification and characterization of HATs and HDACs as writers and erasers was vital for understanding its function. Because some HATs also possess acyltransferase activity (11, 19, 20), we set out to investigate whether the main HATs in yeast, Gcn5 and Esa1, are writers of histone crotonylation in budding yeast. Gcn5 and Esa1 have previously been implicated in histone crotonylation by genetic experiments (20, 21), although neither human GCN5 nor Esa1 and its human homologue MOF exhibited any crotonyltransferase activity *in vitro* (11, 20, 21). Here, we reveal both the *in vitro* and *in vivo* activities of the Gcn5-Ada2-Ada3 (ADA) and Esa1-Yng2-Epl1 (Piccolo NuA4) complexes, thereby identifying these complexes as writers of histone H3 and H4 crotonylation, respectively. Moreover, we show that crotonate treatment triggers a transcription response that depends on Gcn5- and Esa1-dependent histone crotonylation. Thus, the ADA and Piccolo NuA4 complexes exhibit crotonyltransferase activity toward chromatin and are regulators of crotonylation-dependent transcription.

## Results

### Gcn5 is a novel histone crotonyltransferase in vitro

**Figure 1.**
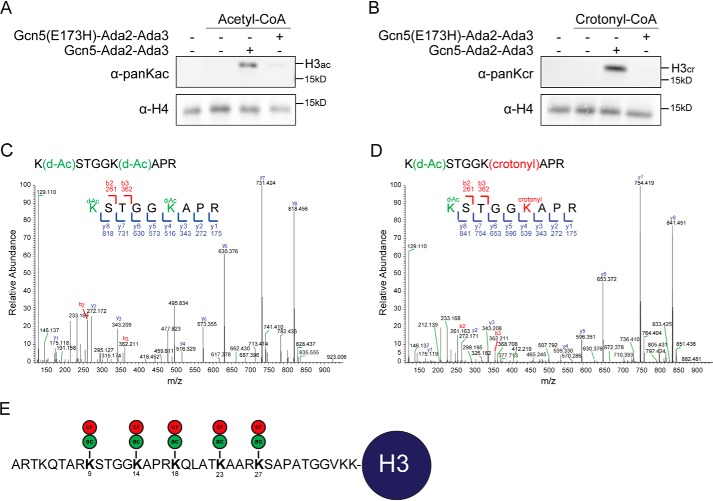
**The ADA complex crotonylates histone H3 in mononucleosomes *in vitro*.** Western blot analysis of *in vitro* (*A*) histone acetyltransferase, (*B*) histone crotonyltransferase reactions with recombinant WT and catalytically-dead (Gcn5-E173H) ADA complex and mononucleosomes as a substrate. *C* and *D,* MS2 spectra of peptides corresponding to a region spanning Lys-9–Arg-17 in histone H3 showing (*C*) unmodified (but deutero-acetylated) K9 and K14 (*green*), (*D*) crotonylated K14 (*red*) with deuterated acetic acid (*d*) acetylations in *green. E,* schematic depicting residues 1–37 of H3 with acetylated (*ac*, *green*) and crotonylated (*cr*, *red*) lysine residues as identified by MS.

Because a role for Gcn5 and Esa1 in crotonylation had not been properly described, we examined whether Gcn5 and Esa1 are *bona fide* writers of this modification in budding yeast. To this end, we first purified Gcn5 from *Escherichia coli* (Fig. S1*A*), and tested whether it can directly crotonylate chromatin *in vitro* using a histone acylation transferase assay. Acetyl-CoA (positive control) and crotonyl-CoA were used as acyl-donors in this assay. The outcome of the reactions was analyzed by Western blot analysis using different pan-K-acyl-specific antibodies. Gcn5 alone, in contrast to the core ADA complex consisting of Gcn5, Ada2, and Ada3, displays weak acetylation activity toward nucleosomes (22, 23), which we confirmed in our assays (Fig. S2*A*). In addition, we also did not observe crotonylation activity (Fig. S2*B*). We therefore purified the core ADA (Gcn5, Ada2, and Ada2) complex from *E. coli* (Fig. S1, *B* and *C*) and tested its activity on histone octamers and on *in vitro* reconstituted mononucleosomes (Fig. S1*D*) (24). As previously shown, the core ADA complex displayed robust acetylation activity mainly toward histone H3 in both histone octamers and nucleosomes (Fig. 1*A,* Fig. S3*A*). This activity depended on the catalytic glutamic acid residue on position 173 in Gcn5, because changing it to histidine (Gcn5-E173H) abrogated acetylation, agreeing with previous reports (Fig. 1*A*) (22, 25). Importantly, the ADA complex also exhibited crotonyltransferase activity toward histone octamers and nucleosomes in a manner dependent on its catalytic activity (Fig. 1, *A* and *B*, and Fig. S3*B*). Thus, we identified the ADA complex as a writer of histone crotonylation *in vitro*.

### Gcn5 targets specific lysines in histone H3 for crotonylation

We then sought to confirm the presence of crotonyl modifications on histone H3 and investigate which lysines in histone H3 are targeted for crotonylation by Gcn5. We therefore performed *in vitro* crotonyltransferase assays using mononucleosomes, separated the reaction products on SDS-PAGE and stained the gel by Coomassie. Bands corresponding to H3 were cut out and samples were analyzed by MS. This revealed that Gcn5 acetylates lysine residues at positions 9, 14, 18, 23, and 27 in histone H3 (Fig. 1, *C–E,* and Fig. S4). Likewise, Gcn5 targeted the same residues for crotonylation (Fig. 1*E*), all of which were known to be acetylated (25). These results were confirmed by Western blot analysis of the *in vitro* reaction products using site-specific antibodies for H3 lysine 9, 14, and 18 (Fig. S5). Of note, two bands were detected, likely representing differently modified H3 species (Fig. S5). We validated these antibodies by Western blot analysis using extracts from yeast cells expressing H3K9A, K3K14A, and H3K18A substitutions. The signal observed in the WT strain was lost in the substitution mutants (Fig. S6, *A–C*), whereas expression of histone H3 was not affected in any of these mutants (Fig. S6*D*) indicating site-specificity of these antibodies. Thus, the ADA complex is capable of directly modifying chromatin by acetylation, as well as crotonylation, targeting the same residues in histone H3.

### Esa1 is a novel histone crotonyltransferase in vitro

**Figure 2.**
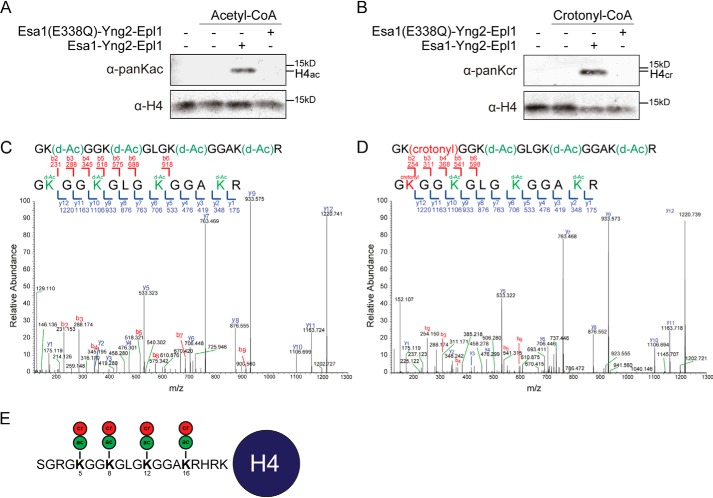
**The Piccolo NuA4 complex crotonylates histone H4 in mononucleosomes *in vitro*.** Western blot analysis of *in vitro* (*A*) histone acetyltransferase, (*B*) histone crotonyltransferase reactions with recombinant WT and catalytically-dead (Esa1-E338Q) Piccolo complex and mono- nucleosomes as a substrate. *C* and *D*, mass spectrometry spectra of peptides corresponding to a region spanning Gly-4–Arg-17 in histone H4 showing (*C*) unmodified (but deutero-acetylated) K5, K8, K12, and K16 (*green*), (*D*) crotonylated K5 (*red*) with deuterated (*d*) acetylations in *green. E,* schematic depicting residues 1–37 of H4 with acetylated (*ac*, *green*) and crotonylated (*cr*, *red*) lysine residues as identified by MS.

Esa1, similar to Gcn5, is unable to acetylate chromatin substrates in the absence of its members in the NuA4 complex (26, 27). In addition, Esa1 alone does not possess any crotonyltransferase activity (20). We therefore purified Esa1 in the presence of Epl1 and Yng2, which are the two other subunits of the Esa1-containing Piccolo NuA4 histone acetyltransferase complex (27) from *E. coli* (Fig. S7, *A* and *B*) (28), and tested its activity toward mononucleosomes *in vitro*. As expected, the Piccolo NuA4 complex displayed robust acetylation activity mainly toward histone H4 (Fig. 2*A*) (23). This activity depended on the catalytic glutamic acid residue on position 338 in Esa1, because changing it to glutamine (Esa1-E338Q) abrogated acetylation, agreeing with previous reports (Fig. 2*A*) (29, 30). The Piccolo NuA4 complex, similar to the ADA complex, also exhibited crotonyltransferase activity toward nucleosomes in a manner dependent on its catalytic activity (Fig. 2*B*). Thus, we identified the Piccolo NuA4 complex as a writer of histone crotonylation *in vitro*.

### Esa1 targets specific lysines in histone H4 for crotonylation

To ascertain the presence of crotonyl modifications on histone H4 and identify the lysines in histone H4 that are crotonylated by Esa1, we examined reaction products from the acyltransferase assay by MS. Similar to the approach for Gcn5, we cut out H4 bands from SDS-PAGE gels and examined the samples by MS. This revealed that Esa1 acetylates lysine residues at positions 5, 8, 12, and 16 in histone H4 for acetylation (Fig. S8), which is in agreement with previous reports (26, 27). Importantly, Esa1 also targeted the same residues for crotonylation (Fig. 2, *D* and *E*, Fig. S8). Therefore, the Piccolo NuA4 complex is capable of directly modifying chromatin by acetylation, as well as crotonylation, targeting the same residues in histone H4.

### Metabolic regulation of crotonylation is Gcn5- and Esa1-dependent

**Figure 3.**
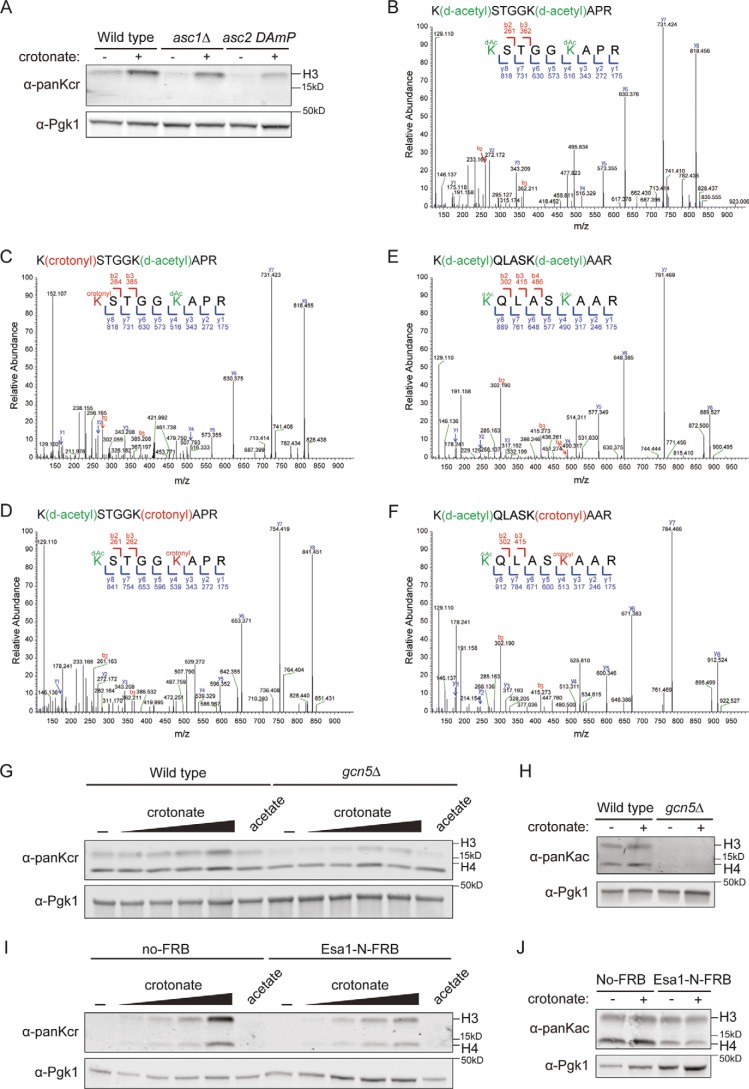
**Crotonate affects histone crotonylation in Gcn5- and Esa1-dependent manner.** *A,* WT, *acs1*Δ and *acs2_DAmP* strains were treated with sodium crotonate (10 mm, pH 7.5) for 3.5 h in the presence of 0.8 m sorbitol. Whole cell extracts were immunoblotted with the indicated antibodies. *B–F,* MS2 spectra of peptides corresponding to a region spanning Lys-9–Arg-17 in histone H3 showing (*B*) unmodified (but deuteroacetylated) K9 and K14 (*green*), (*C*) crotonylated K9 (*red*), and (*D*) crotonylated K14 (*red*). *E* and *F,* MS2 spectra of peptides corresponding to a region spanning Lys-18–Arg-21 in histone H3 showing (*E*) unmodified (but deuteroacetylated) K18 and K23 (*green*) and (*F*) crotonylated K23 (*red*). *G* and *H*, WT and *gcn5*Δ strains were treated with (*G*) increasing concentrations of sodium crotonate (1, 2, 5, and 10 mm, pH 7.5) or sodium acetate (10 mm, pH 7.5) or (*H*) sodium crotonate (10 mm, pH 7.5) for 3.5 h in the presence of 0.8 m sorbitol. Whole cell extracts were immunoblotted with the indicated antibodies. *I* and *J*, nontagged (no FRB) and Esa1-N-FRB tagged strains were treated as in *G and H* in the presence of rapamycin. Whole cell extracts were immunoblotted with the indicated antibodies.

Following our *in vitro* studies on the role of Gcn5 and Esa1 in crotonylation, we sought to address the relevance of these Gcn5-dependent and Esa1-dependent modifications *in vivo*. To this end, we added external sodium crotonate to the yeast medium. This compound can be ligated into the corresponding acyl-CoAs, as previously shown in human cells (11). Indeed, crotonate addition increased crotonylation on histone H3 (Fig. 3*A*) and this was largely dependent on the presence of the yeast acetyl-CoA synthetase enzymes Acs1 and Acs2 (Fig. 3*A*). To further study these crotonate-induced changes, we performed Western blot analysis of H3K9cr H3K14cr and H3K18cr in yeast chromatin following crotonate treatment (Fig. S6). Importantly, whereas the signals for these marks moderately increased following crotonate treatment of WT cells, the H3K9cr, H3K14cr and H3K18cr signals were lost in yeast cells expressing the H3K9A, H3K14A and H3K18A substitutions (Fig. S6). This finding suggests site-specific crotonyl recognition of these antibodies under increased crotonate conditions. To corroborate these findings, we also examined crotonylation in chromatin from WT yeast cells by MS. As a control, we included chromatin from HeLa cells, in which the relative abundances of H3K14ac and H3K14cr was around 8 and 0.02%, respectively (31). In WT yeast that were treated with crotonate or left untreated, we did not detect crotonylation on the histone tails of H4, indicative of low abundance of this modification. In accordance with these findings, crotonylation could also not be detected on the histone tails of H4 from HeLa cells (17). However, we identified crotonylation on the N-terminal tail of H3 in chromatin from crotonate-treated WT yeast cells. Specifically, we found lysine 9, 14, and 23 to be crotonylated (Fig. 3, *B–F*). These lysines were among the residues targeted by Gcn5 crotonylation *in vitro* (Fig. 1, Fig. S4), indicating a Gcn5-dependent crotonate response that involves H3 crotonylation *in vivo*.

To examine whether Gcn5 and Esa1 are responsible for crotonylation *in vivo*, we examined crotonate-induced crotonylation in the absence of these enzymes. We observed a dose-dependent increase in histone crotonylation, which was reduced in the absence of Gcn5 (Fig. 3*G*, Fig. S9). Because Esa1 is an essential gene, we employed the anchor-away technique to rapidly deplete FRB-tagged Esa1 from the nucleus following rapamycin treatment (32). As expected, rapamycin treatment severely reduced histone acetylation, although levels in the nontagged/no-FRB strain remained unaffected (Fig. S10). Importantly, crotonate-induced histone crotonylation was also reduced in the rapamycin-treated Esa1-N-FRB strain (Fig. 3*I*, Fig. S9). We did not observe cross-talk between crotonylation and acetylation because crotonylation was not affected by the addition of acetate (Fig. 3, *G* and *I*), which is known to affect acetylation (33). Likewise, adding crotonate to the medium did not lead to a change in the acetylation levels (Fig. 3, *H* and *J*). Similarly, changes in the abundance of succinyl-CoA did not affect acetylation levels (19). We therefore conclude that histone crotonylation responds to metabolic changes in crotonyl-CoA abundance in a manner dependent on Gcn5 and Esa1.

### Gcn5 and Esa1 mediate crotonylation-dependent transcription

**Figure 4.**
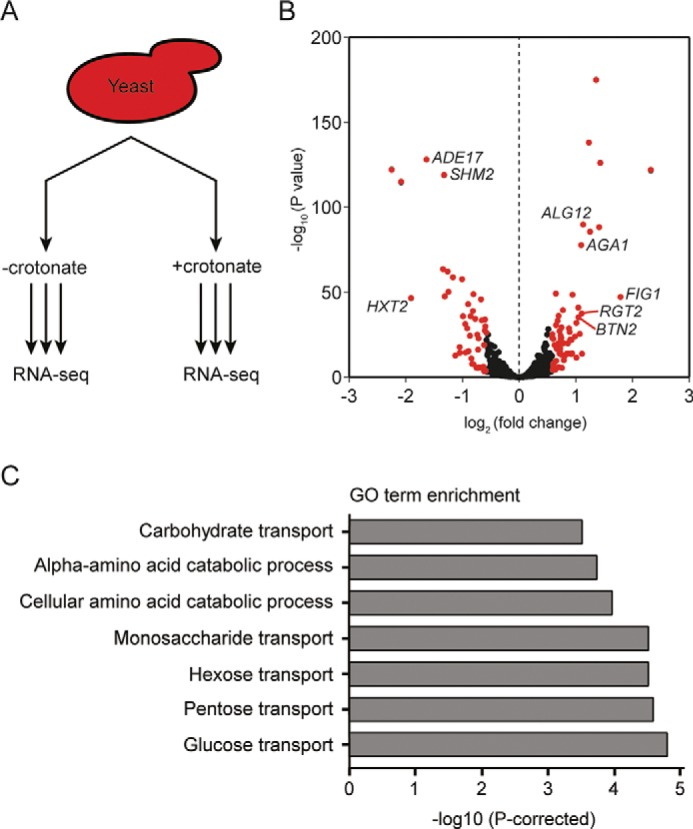
**Crotonate induces various transcription changes.** *A,* schematic of RNA-Seq analysis performed in triplicate on WT yeast strains treated with 10 mm crotonate for 3.5 h. *B,* volcano plot depicting crotonate-induced transcriptome changes. Genes showing a >1.5-fold change in expression (*n* = 106) are highlighted in *red*, genes tested for follow-up are identified in the plot. *C*, GO term analysis of significantly changed genes.

**Figure 5.**
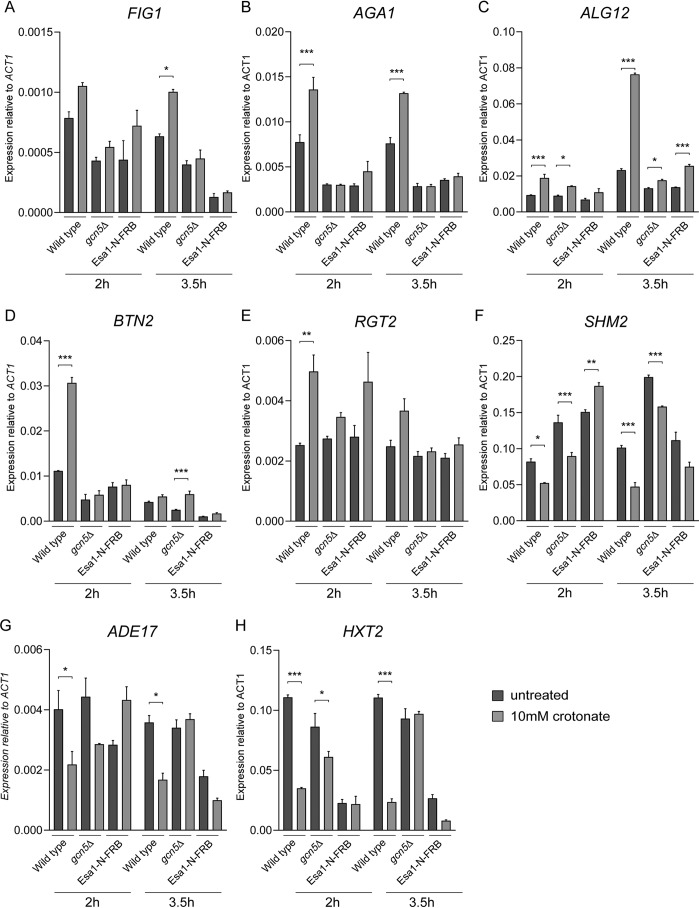
**Crotonate-induced transcription depends on Gcn5 and Esa1.** *A–H*, RT-qPCR analysis of crotonate-treated WT, *gcn5*Δ, and Esa1-N-FRB cells. The 2-ΔΔ*C_t_* relative quantitative method was used to analyze the level of mRNA expression compared with untreated WT cells. Expression was normalized to *ACT1*. Data are represented as the mean fold-change in relative expression due to crotonate addition from at least three independent experiments mean ± S.E.

To investigate the relevance of metabolic regulation of crotonylation, we decided to study its effect on transcription. WT cells were grown in the absence or presence of crotonate for 3.5 h and mRNA was isolated and deep-sequenced (Fig. 4*A*). We identified 880 transcripts that were differentially expressed following crotonate treatment (*p* < 0.05). In contrast to a previous report suggesting that crotonylation has a stimulatory effect on transcription (11, 34), we found that transcript levels were both increased and decreased (Table S1, Fig. 4*B*). This may suggest that crotonylation can both promote and suppress transcription. A GO term analysis of the differentially expressed genes (>1.5-fold change) revealed that crotonate mainly influenced the expression of genes involved in glucose (*p* = 0.000016) and pentose transport (*p* = 0.000027) (Fig. 4*B*, Table S1), several of which (*e.g. HXT1*, *HXT2*, *HXT4*, *HXT7*, *HXK1*, and *RGT2*) respond similarly to high glucose levels (35) (Table S1). Importantly, for a selection of genes we could confirm the observed transcriptional changes by using reverse transcription (RT)-qPCR (Fig. 5). Because we found that crotonate-induced crotonylation relies on Gcn5 and Esa1 (Fig. 5), we next asked if the crotonate-induced transcriptional changes also depend on Gcn5 and Esa1. Indeed, RT-qPCR showed that the up- and down-regulation of the selected genes was determined by either Gcn5 and/or Esa1 (Fig. 5). To exclude indirect effects of crotonate treatment on gene expression, we investigated if the crotonate-induced transcriptional changes also appear at an earlier time point (Fig. 5). Indeed, transcriptional changes already appeared after 2 h of crotonate treatment and were also dependent on Gcn5 and Esa1. This demonstrates that the metabolic state of cells controls gene transcription in a manner dependent on Gcn5's and Esa1's crotonyltransferase activity.

### Gcn5 and Esa1 are recruited to a subset of crotonate-induced genes

**Figure 6.**
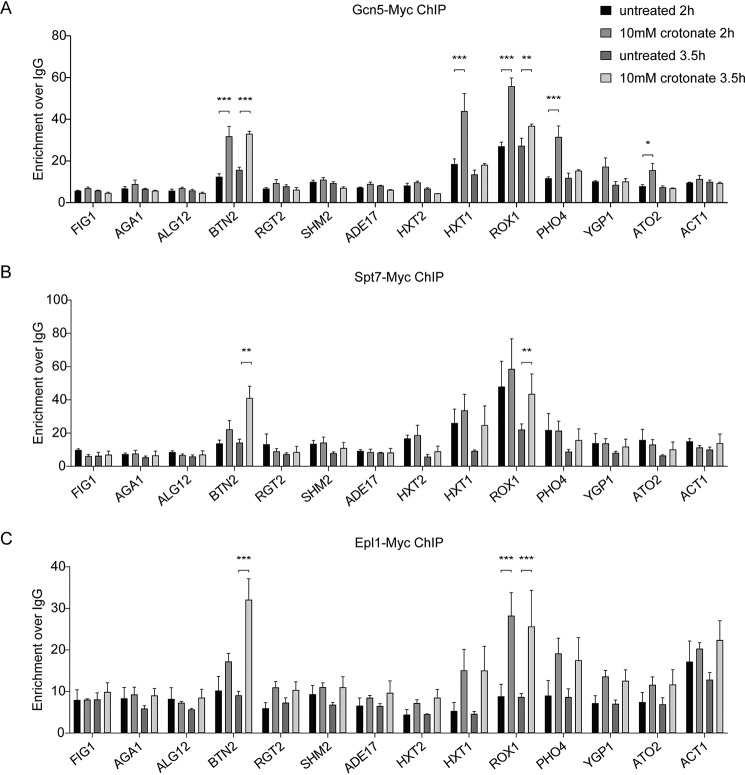
**Crotonate-dependent recruitment of Gcn5, Spt7, and Epl1 to gene promoters.** ChIP-qPCR analysis of (*A*) Gcn5-Myc, (*B*) Spt7-Myc, and (*C*) Epl1-Myc at gene promoters before and after crotonate treatment. Data represent the mean fold-enrichment over an IgG control ChIP from three independent experiments mean ± S.E.

To investigate the direct effect of the ADA and Piccolo NuA4 complexes on crotonate-induced transcriptional changes, we studied their occupancy at promoters of genes that are regulated by crotonate. Although both Gcn5 (ADA complex) and Epl1 (Piccolo NuA4 complex) were not recruited to the down-regulated genes *ADE17*, *SHM2*, and *HXT1* at 2 and 3.5 h after crotonate treatment, we observed recruitment to a subset of the up-regulated genes upon stimulation with crotonate (Fig. 6, *A* and *C*). In addition, the SAGA subunit Spt7 was also recruited to both *BTN2* and *ROX1* upon crotonate stimulation (Fig. 6*B*), suggesting that Gcn5 regulates crotonate-induced transcriptional as the catalytic subunit of both the ADA and SAGA complexes. To rule out that the transcriptional changes were induced by Gcn5- and Esa1-dependent acetylation, we monitored the acetylation status of several gene promoters following crotonate stimulation. Importantly, no major crotonate-induced changes in the acetylation levels were observed at these promoters (Fig. S11). Taken together, these results suggest that the ADA/SAGA and Piccolo NuA4 complexes are able to directly regulate crotonate-induced transcription independently of histone acetylation.

## Discussion

Here, we found that the ADA and Piccolo NuA4 complexes exhibit crotonylation activities *in vitro*, revealing these complexes as *bona fide* acylation writers. Previously, it was reported that neither KAT2A (hGCN5) nor its homologue P300/CBP-associated factor (PCAF) possesses crotonylation activity *in vitro* (11). Although Gcn5 and KAT2A may display the same functionality, the lack of ADA subunits in the *in vitro* assays could explain the lack of activity. Indeed, both Gcn5 and KAT2A were shown to display reduced acetyltransferase activity in the absence of Ada2 and Ada3 (22, 36). Similarly, Esa1 requires the presence of its NuA4 complex members, Yng2 and Epl1, to crotonylate (Fig. 2*B*). In addition, the activity of various HATs toward longer acyl-CoA chains, such as crotonyl-CoA or succinyl-CoA, was found to be decreased (17, 37, 38). Similarly, Gcn5 and Esa1 may also function as weak crotonyltransferases, even in the context of the ADA and Piccolo NuA4 complexes. Such scenarios would be in accordance with the limited detection of histone crotonylation by MS and the generally lower abundance of nonacetyl acylations compared with acetylation (17). Although loss of Gcn5 or Esa1 substantially decreased crotonylation levels, in their absence, crotonate-induced crotonylation was not fully inhibited (Fig. 3, *G–I,* Fig. S9). Therefore, other enzymes may partly compensate Gcn5 or Esa1 crotonyltransferase activities.

Because both Gcn5 and Esa1 recognize various acyl-CoAs and function as broad acyltransferases, it is challenging to distinguish the effects of specific acylation modifications on cellular processes. For p300 and KAT2A, which also recognize multiple acyl-CoAs, separation-of-function mutants were constructed to eliminate broad substrate specificity and generate substrate-specific acyltransferases (19, 20). An acetylation-deficient, but crotonylation-proficient p300 mutant remained competent in promoting transforming growth factor-β1–induced gene activation, attributing a role for crotonylation in transcriptional regulation (20). Expression of a succinylation-deficient, but acetylation-proficient KAT2A mutant inhibited tumor growth in mice, demonstrating that succinylation has nonredundant roles to acetylation (19). By analogy, identifying separation-of-function mutants for Gcn5 or Esa1, which are defective in crotonylation yet proficient in generating other acylation modifications, would enable genetic studies on Gcn5- or Esa1-induced crotonylation and its contribution to cellular processes.

Here we modulated crotonylation at the metabolic level by exposing cells to crotonate. The subsequent increase in histone crotonylation invoked various transcriptional changes, particularly in metabolic pathways related to glucose transport, in a manner dependent on Gcn5 and/or Esa1. Considering increased crotonylation mimics certain transcriptional changes seen under high glucose levels, crotonyl-CoA levels could be influenced by glucose availability, as described for acetyl-CoA (33). Similarly, high glucose increases Gcn5-dependent acetylation and enhances recruitment of Gcn5 to promoters of genes involved in growth regulation (39–41). We infer that Gcn5 and Esa1 may couple the metabolic state of cells to transcriptional changes through various histone acylation modifications, in line with previous reports that link the availability of acyl-CoA metabolites to their corresponding histone acylation levels (17).

Several genes were regulated by crotonate treatment of cells. To assess whether these genes were regulated by promoter crotonylation, we examined the recruitment of several subunits of the crotonyl writer complexes ADA/SAGA (Gcn5 and Spt7) and Piccolo NuA4 (Epl1) as a proxy for crotonylation at these promoters. Indeed, these complexes were recruited to promoters of a subset of the crotonate-induced genes, suggesting they play a direct role in regulating crotonylation-dependent transcription. This was further corroborated by the fact that acetylation levels remained unaffected at these promoters. On the other hand, the subset of gene promoters at which we did not observe ADA/SAGA (Gcn5 and Spt7) and Piccolo NuA4 (Epl1) accumulation are likely regulated through secondary transcriptional changes induced by crotonate treatment. These changes could be Gcn5 and Esa1-dependent and -independent.

Because Gcn5 and Esa1 generate multiple acyl modifications on chromatin, specificity in the transcriptional response has to come from other factors that act downstream of Gcn5 and Esa1. Downstream of acyl writer proteins, acyl-specific reader proteins such as YEATS domain proteins or double PHD finger proteins have a higher affinity for crotonylation compared with acetylation (42, 43), and can therefore compete for acylation binding (13, 44–46). YEATS domain proteins, such as the reader protein Taf14, could induce the distinct transcriptional changes evoked by changes in histone crotonylation through Gcn5 or Esa1. Further studies are required to better understand the mode-of-action of these proteins in “reading” distinct acylation modifications during transcription regulation and other yet-to-be identified acylation-dependent processes.

## Experimental procedures

### Yeast strains, cell lines, plasmids and antibodies

Yeast strains and plasmids used in this study are listed in Tables 1 and 2. pRS315-GCN5 plasmid was obtained from Dr. Paul van Heusden (Leiden University, The Netherlands) (47). Histone H3 and H4 mutant collection was purchased from Dharmacon. Human HeLa cells were STR genotypes and cultured in 5% CO_2_ at 37 °C in Dulbecco's modified Eagle's medium supplemented with 10% fetal calf serum and antibiotics. No-FRB and Esa1-N-FRB strains were constructed based on conventional PCR-based transformation protocols. Detailed construction information is available upon request. For N-terminal tagging of the Esa1 protein, 500 bp upstream and 500 bp downstream sequences from the start codon were inserted in pFA6a-KanMX6 plasmid (48) after the KanMX6 cassette using Gibson assembly cloning (New England Biolabs, E2611). The FRB tag (32) was then inserted after the start codon using Gibson assembly cloning. Cells were transformed with PCR product obtained by PCR on the resulting plasmid using forward primer (40 bp before Esa1 start codon + GAATTCGAGCTCGTTTAAAC) and reverse primer (∼20 bp reverse complement sequence before position +500 bp) and selected for G418 resistance. The region corresponding to the N-terminal region of the protein was sequence verified. The yeast strain used for transformation was previously mutated (*tor1-1* mutation and *FPR1* deletion) to make the cells resistant to rapamycin, while Rpl13a was tagged with FKBP12, as described (32). The pST44-yAda3t2HISx3-yAda2 × 3-yGcn5 × 5 polycistronic vector containing yeast *GCN5*, *ADA2*, and *ADA3* was provided by Dr. Sean Taverna (Johns Hopkins University School of Medicine) (25). The pREST B-GCN5 plasmid was provided by Dr. Min-Hao Kuo (Michigan State University) (49). The pST44-yEpl1A3 × 3-Yng2A1 × 3-HISyEsa1 polycistronic vector containing yeast *ESA1*, *YNG2*, and *EPL1* was provided by Dr. Song Tan (The Pennsylvania State University) (28). The catalytically-dead Gcn5-E173H mutant was generated by site-directed mutagenesis of pST44-yAda3t2HISx3-yAda2 × 3-yGcn5 × 5, whereas the catalytically-dead Esa1-E338Q mutant was generated by site-directed mutagenesis of pST44-yEpl1A3 × 3-Yng2A1 × 3-HISyEsa1. Antibodies used were anti-pan-acetylation (PTM-105; PTM-Biolabs), anti-pan-crotonylation (PTM-501; PTM Biolabs), anti-H3K9cr (PTM-516; PTM Biolabs), anti-H3K14cr (GC-545; PTM Biolabs), anti-H3K18cr (PTM-517; PTM Biolabs), anti-Pgk1 (number 459250; Invitrogen), anti-H3 (ab1791; Abcam), anti-H4ac (06-946, penta; Upstate), and anti-H4 (ab7311; Abcam).

**Table 1:** **Yeast strains**

Name	Genotype	Reference
yHA-829 wildtype	*MAT*a *can1*Δ*-STE2pr-Sphis5 lyp1*Δ *his3*Δ*1 leu2*Δ*0 ura3*Δ*0 met15*Δ*0 ho*Δ *(Barcoder001)KanMX*	NKI 4560 (53)
yHA-1028 *gcn5*Δ	*MAT*a *can1*Δ*-STE2pr-Sphis5 lyp1*Δ *his3*Δ*1 leu2*Δ*0 ura3*Δ*0 met15*Δ*0 ho*Δ*(Barcoder)KanMX gcn5*Δ*::NatMX*	54
Histone H3 and H4 mutant collection	*MATa his3*Δ*200 leu2*Δ*0 lys2*Δ*0 trp1*Δ*63 ura3*Δ*0 met15*Δ0 can1::MFA1pr-HIS3 hht1-hhf1::NatMX4 hht2-hhf2::[HHTS-HHFS]*-*URA3*	55
*acs1*ΔNatMX collection	*MAT*a *can1*Δ*-STE2pr-Sphis5 lyp1*Δ *his3*Δ*1 leu2*Δ*0 ura3*Δ*0 met15*Δ*0 ho*Δ*(Barcoder)KanMX acs1*Δ*::NatMX*	54
*Acs2 DaMP* NatMX DaMP collection	*MAT*α *can1*Δ*-STE2pr-Sphis5 lyp1*Δ*-STE3pr-LEU2 his3*Δ*1 leu2*Δ*0 ura3*Δ*0 met15*Δ*0*	56
QY3530a *No-FRB*	*MATa ho hml::ADE1 hmr::ADE1 ade1–100 leu2–3,112 lys5 trp1::hisG ura3–52 ade3::GAL10::HO tor1–1::URAMX fpr1*Δ::HphMX Rpl13A-2*FKBP12::TRP	This study
QY3562 *RPO21-FRB*	Isogenic to QY3530 except *RPO21-FRB::KanMX*	This study
QY3572a *ESA1-N-FRB*	Isogenic to QY3530 except *ESA1-FRB::KanMX*	This study
QY237 *EPL1–13MYC*	*MATa his3*Δ*1 leu2*Δ*0 ura3*Δ*0 met15*Δ*0 EPL1–13MYC::HIS3*	57
QY3686 *SPT7-MYC*	*MATa ho hml::ADE1 hmr::ADE1 ade1–100 leu2–3,112 lys5 trp1::hisG ura3–52 ade3::GAL10::HO SPT7–13MYC::TRP*	This study
yHA-1078 *GCN5-MYC*	Isogenic to yHA-829 except *GCN5-MYC::HpH*	This study
yHA-1079 *YAF9-MYC*	Isogenic to yHA-829 except *YAF9-MYC::HpH*	This study

**Table 2:** **Plasmids**

Name	Plasmid	Reference
pHA-434	pST44-yAda3t2HISx3-yAda2 × 3-yGCN5 × 5	25
pHA-485	pST44-yAda3t2HISx3-yAda2 × 3-yGCN5(E173H)x5	This study
pHA-431	pRSET–6xHis-T7-GCN5	49
pHA-436	pST44-yEpl1A3 × 3-Yng2A1 × 3-HISyEsa1	28
pHA-700	pST44-yEpl1A3 × 3-Yng2A1 × 3-HISyEsa1(E338Q)	This study

### Recombinant protein purification

BL21-CodonPlus (DE3)-RIL *E. coli* cells (Agilent) were transformed with pST44-yAda3t2HIS × 3-yAda2 × 3-yGcn5 × 5 or pST44-yEpl1A3 × 3-Yng2A1 × 3-HISyEsa1 plasmids and grown overnight in the presence of 1 mm isopropyl 1-thio-β-d-galactopyranoside. Cell pellets were lysed (50 mm Tris, pH 7.5, 500 mm NaCl, 10% glycerol, BugBuster 1x (Merck), 1 mm MgCl_2_, 10 mm β-mercaptoethanol, 15 kilounits of lysozyme, 250 units of benzonase, 1 tablet protease inhibitors (Sigma, 1 tablet cOmplete^TM^, Mini, EDTA-free Protease Inhibitor Mixture Protease Inhibitor Mixture)), and sonicated. Salt concentration in the supernatant was lowered to 100 mm NaCl by adding 50 mm Tris, pH 7.5, 10% glycerol, and 10 mm β-mercaptoethanol. The lysate was loaded on an ÄKTA Pure Chromatography System on which a 1-ml HiTrapQ HP column was installed (GE Healthcare). The column was washed with 5 column volumes of wash buffer (20 mm Tris, pH 8, 100 mm NaCl, 10% glycerol, 10 mm β-mercaptoethanol). Elution was performed by raising the NaCl concentration to 500 mm in the wash buffer and using a 20-column volume linear gradient while collecting 2-ml fractions. Gcn5-Ada2-Ada3 or Esa1-Yng2-Epl1 complexes were purified on nickel beads using C terminally His-tagged Ada3 or Esa1. Nickel bead suspension (Qiagen) was added to the tubes and incubated for 1 h at 4 °C. Beads were washed 3 times and proteins were eluted 3 times in 200 μl elution buffer (50 mm Tris, pH 7.5, 500 mm NaCl, 10% glycerol, 300 mm imidazole). Eluates were transferred to 100-kDa Vivaspin 500 filter cups (Sartorius) to lower the imidazole concentration and concentrate the purified complexes. The residual was snap frozen in liquid nitrogen and stored at −80 °C.

### Nucleosome reconstitution

Purified histones and mononucleosomes were isolated as described (24, 50).

### In vitro acylation assays

Histone acylation assays were performed with recombinant WT Gcn5-Ada2-Ada3 complex, catalytically-dead Gcn5(E173H)-Ada2-Ada3 complex, WT Esa1-Yng2-Epl1, and catalytically-dead Esa1(E338Q)-Yng2-Epl1 using 0.5 μm histone octamers and 30 μm acetyl-CoA, 300 μm butyryl-CoA, 300 μm crotonyl-CoA, or 300 μm succinyl-CoA in reaction buffer (50 mm Tris-Cl, pH 7.5; 100 mm NaCl; 1 mm EDTA, 1 mm DTT) with a final volume of 20 μl. Mononucleosomes were used at a concentration of 0.5 μm. Reactions were performed for 1 h at 30 °C. Reactions were inhibited by adding sample buffer and analyzed by Western blotting. For MS, samples were separated on SDS-PAGE, the gel was stained with Coomassie, and bands corresponding to H3 and H4 were excised from the gel for analysis.

### Mass spectrometry

Gel slices containing histone H3 or H4 protein were subjected to reduction with DTT and alkylation with iodoacetamide using Proteineer DP digestion robot (Bruker). Next, lysines were modified with acetic acid anhydride to block excessive tryptic cleavage of the arginine- and lysine-rich histone proteins. We used deuterated acetic acid anhydride to discriminate between acetylation by the Gcn5-Ada2-Ada3 and Esa1-Yng2-Epl1 complexes and the blocking agent. Slices were incubated for 1 h at room temperature in 20% (v/v) acetic anhydride-*d*_6_ (Sigma-Aldrich, 175641), 60% (v/v) methanol, and 10 mm NH_4_HCO_3_, pH 8.4. Next, they were washed three times with acetonitrile and 10 mm NH_4_HCO_3_, pH 8.4, prior to protein digestion with trypsin. Peptides were lyophilized, dissolved in 95/3/0.1 (v/v/v), water/acetonitrile/formic acid, and subsequently analyzed by on-line C18 nanoHPLC MS/MS with a system consisting of an Easy nLC 1200 gradient HPLC system (Thermo, Bremen, Germany), and a LUMOS mass spectrometer (Thermo). Fractions were injected onto a homemade precolumn (100 μm × 15 mm; Reprosil-Pur C18-AQ 3 μm, Dr. Maisch, Ammerbuch, Germany) and eluted via a homemade analytical nano-HPLC column (30 cm × 50 μm; Reprosil-Pur C18-AQ 3 μm). The gradient was run from 10 to 40% in solvent B (20/80/0.1, water/acetonitrile/formic acid (FA) (v/v)) for 30 min. The nano-HPLC column was drawn to a tip of ∼5 μm and acted as the electrospray needle of the MS source. The LUMOS mass spectrometer was operated in data-dependent MS/MS mode for a cycle time of 3 s, with a HCD collision energy at 32 V, and recording of the MS2 spectrum in the Orbitrap. In the master scan (MS1) the resolution was 120,000, the scan range was 400–1500, at an AGC target of 400,000 at a maximum fill time of 50 ms. Dynamic exclusion after *n* = 1 with exclusion duration of 10 s. Charge states 2–5 were included. For MS2 precursors were isolated with the quadrupole with an isolation width of 1.2 Da. First mass was set to 110 Da. The MS2 scan resolution was 30,000 with an AGC target of 50,000 at a maximum fill time of 60 ms.

In a post-analysis process, raw data were first converted to peak lists using Proteome Discoverer version 2.2.0.388 (Thermo Electron), and then submitted to the Uniprot database (452772 entries) using Mascot version 2.2.04 (Matrix Science) for protein and peptide identification. Mascot searches were with 10 ppm and 0.02 Da deviation for precursor and fragment mass, respectively, and trypsin as enzyme with up to two missed cleavages allowed. Methionine oxidation and *d*_6_-acetylation, acetylation, and crotonylation of lysines were set as variable modification. Carbamidomethyl on cysteine was set as a fixed modification. The false discovery rate was set to 1% and the Mascot ion threshold score to 35. All reported acetylated and crotonylated spectra were also inspected manually. Peptides are listed in Table S2. Mass spectrometry data have been uploaded to the PRIDE repository (accession number PXD015488).

### RNA-Seq

Three independent yeast colonies were used for inoculation. Overnight cultures were diluted in fresh medium with or without 10 mm sodium crotonate (Sigma-Aldrich) in the presence of 0.8 m sorbitol (Sigma-Aldrich). After 3.5 h cells were in early log-phase (1 × 10^7^ cells/ml) and harvested by centrifugation. Total RNA was isolated using the RNeasyMini kit (Qiagen) and treated with the RNase-free DNase Set (Qiagen) to remove any contaminating genomic DNA. Library preparations were performed with the TruSeq Stranded mRNA Library kit (Illumina). Three independent biological replicates for each condition were subjected to RNA-seq analysis using an Illumina NextSeq500 with a 75-bp single read flowcell. The raw source data files are available via ArrayExpress, accession number E-MTAB-7471. Reads were aligned to the UCSC SacCer3 reference genome (April 2011) using Tophat20 with the following parameters “-G SacCer3_SGD.gtf-I 1000-i 20-p 6-o,” and counted with HTSeq with the following parameters “htseq-count-f bam-stranded = yes.” Differential expression was analyzed in *R* by DESeq2 (51) using the default parameters, including the Benjamini-Hochberg procedure for adjusting *p* values for multiple testing by converting them to *padj*. GO term analysis was performed with the GO Term Finder (version 0.86) of the Saccharomyces Genome Database (www.yeastgenome.org).^3^

### RT-qPCR

Total RNA was isolated using the RNeasy Mini kit (Qiagen) and treated with the RNase-free DNase Set (Qiagen) to remove any contaminating genomic DNA. cDNA was prepared from total RNA using the GoScript^TM^ Reverse Transcription System according to the manufacturer's protocol (Promega). qPCR was performed using GoTaq qPCR Master Mix (Promega). Expression levels were quantified using the 2-ΔΔ*C_t_* method with *ACT1* as a reference. Specificity of primers was confirmed by melting curve analysis and tested for linear amplification. All primers used in qPCR are listed in Table 3.

**Table 3:** **Primers**

Number	Name	Sequence
oHA-1984	GCN5 E173H F	GACCTTTCGATAAGAGAGAGTTCGCACATATTGTTTTCTGTGCCATCAG
oHA-1985	GCN5 E173H R	TGCGAACTCTCTCTTATCGAAAGGTCGATATGTTATGCCACCTACG
oHA-2278	ESA1 E338Q F	CAAAGTTGGTTCTCCCCAGAAACCTTTGTCTG
oHA-2279	ESA1 E338Q R	CAGACAAAGGTTTCTGGGGAGAACCAACTTTG
oHA-3211	FIG1 RT-qPCR F	GATCCGGTTACATGGGTGTT
oHA-3212	FIG1 RT-qPCR R	GACAACGCTTGATTGGGTTT
oHA-3213	AGA1 RT-qPCR F	TCAAGCTGGCACTACGACAT
oHA-3214	AGA1 RT-qPCR R	AAGGACTTATTTCGGCAGCA
oHA-3215	ALG12 RT-qPCR F	GAATGGTGTCTACCTGAG
oHA-3216	ALG12 RT-qPCR R	AAGTAATGCGTGAAATAAG
oHA-3217	BTN2 RT-qPCR F	ACTTTGGATTTCTCTGTGTCTGG
oHA-3218	BTN2 RT-qPCR R	CTGCTTAGGGACTCGTTGTATC
oHA-3219	RGT2 RT-qPCR F	GCCTACGACAGAGGGAAGAA
oHA-3220	RGT2 RT-qPCR R	CTGTGTATGCATTGCGGTGT
oHA-3221	ADE17 RT-qPCR F	TATCACACATGCCCCAGAAA
oHA-3222	ADE17 RT-qPCR R	CCCTAGCAAGGATACCACCA
oHA-3223	SHM2 RT-qPCR F	AAAGGGTGTTGATGGTGCTC
oHA-3224	SHM2 RT-qPCR R	CACCTGGAACCAAAGCAGAT
oHA-3225	HTX2 RT-qPCR F	TCGTGCTATGGCTATTGCTG
oHA-3226	HTX2 RT-qPCR R	ACCAAACAGCCCATGAAGAC
oHA-3227	ACT1 RT-qPCR F	ACGTTCCAGCCTTCTACGTTTCCA
oHA-3228	ACT1 RT-qPCR R	TCGAAGTCCAAGGCGACGTAACAT
oHA-3229	ATO2 ChIP-qPCR F	GGAACGGCTCCATCCTAAAT
oHA-3230	ATO2 ChIP-qPCR R	CCAGAAATGAGGAGACTGTTGA
oHA-3231	YGP1 ChIP-qPCR F	AGAGGCTCAGGAGCTGAAA
oHA-3232	YGP1 ChIP-qPCR R	TGTTGAACTGTAGCATCGAGAAG
oHA-3233	RGT2 ChIP-qPCR F	CTCTGCTGTGGTTCCTCTTTATC
oHA-3234	RGT2 ChIP-qPCR R	TGGAATGAGTTCCTTGCGATAC
oHA-3235	ROX1 ChIP-qPCR F	CTTGGCGATTGCTGACAAAG
oHA-3236	ROX1 ChIP-qPCR R	GGCAAGACAATACGAGGAAGA
oHA-3237	HXT2 ChIP-qPCR F	GACTTCTTCTCCTCCCACAAA
oHA-3238	HXT2 ChIP-qPCR R	TCTCCGGATTCATAAGGATTAGC
oHA-3239	PHO4 ChIP-qPCR F	GCCGTAGGTACGTTCATTTCTA
oHA-3240	PHO4 ChIP-qPCR R	GCACTTGCAGCATAGGAAATAC
oHA-3241	HXT1 ChIP-qPCR F	GAAGTGACCCAGTGCTCTTT
oHA-3242	HXT1 ChIP-qPCR R	CCTGCAGCATTTCTTCCTAGT
oHA-3243	SHM2 ChIP-qPCR F	CTGCCCACACTGCTTATAGT
oHA-3244	SHM2 ChIP-qPCR R	GACTCGACCGACTCTGTTTC
oHA-3245	ACT1 ChIP-qPCR F	GATGAAGCTCAATCCAAGAGAGG
oHA-3246	ACT1 ChIP-qPCR R	AGTTGGTGGAGAAAGAGTAACCACG
oHA-3247	ADE17 ChIP-qPCR F	ACTGCCACCGGTAAGAAATC
oHA-3248	ADE17 ChIP-qPCR R	CTATACCTCCATAACGACAGAGTATTT
oHA-3249	AGA1 ChIP-qPCR F	TCCCTACATGCTTCGGAATTT
oHA-3250	AGA1 ChIP-qPCR R	GGTGGCTGCTGAGATCTTTA
oHA-3251	FIG1 ChIP-qPCR F	TCCAAGAATGCCGGTGATATG
oHA-3252	FIG1 ChIP-qPCR R	AGGCAATGCAGGGTGATTT
oHA-3253	BTN2 ChIP-qPCR F	AGCATGCTGTAGTGTATGTACTG
oHA-3254	BTN2 ChIP-qPCR R	ACAGACGGTGCGAAAGAAA
oHA-3255	ALG12 ChIP-qPCR F	CCACTTGCTTGTTTACCCTTTC
oHA-3256	ALG12 ChIP-qPCR R	ACTACCCTGCCTCGTAACA

### Western blot analysis

Whole cell extracts were prepared from ∼2.5 × 10^7^ cells. Cell pellets were incubated for 10 min in 1.85 n NaOH and 7.4% β-mercaptoethanol, followed by precipitation with trichloroacetic acid (TCA). Samples were dissolved and boiled in SDS-PAGE sample. Proteins were resolved in 12% polyacrylamide gels and transferred onto polyvinylidene difluoride membranes. Membranes were blocked with blocking buffer (Rockland) in PBS followed by overnight incubation with primary antibody in blocking buffer at 4 °C. Membranes were washed with 0.1% Tween 20 in PBS. Secondary antibody incubations were performed for 60 min in blocking buffer at room temperature using LI-COR® Odyssey IRDye® 800CW (1:10,000). Membranes were subsequently scanned on a LI-COR Odyssey® IR Imager (Biosciences).

### Spot dilution assays

Serial dilutions (10-fold) of mid-log phase cells were spotted on YPD plates and grown for 2–3 days at 30 °C.

### ChIP-qPCR

ChIP was performed as described previously (52). Input and immunoprecipitated DNA was analyzed by qPCR using primers listed in Table 3.

### Statistical analysis

Statistical analysis of RT-qPCR and ChIP-qPCR experiments was performed using two-way analysis of variance with correction for multiple comparisons using the Šídák method in Prism8 software. Significance is indicated as: ***, *p* < 0.001; **, *p* < 0.01; *, *p* < 0.05; *ns*, not significant. All error bars represent the mean ± S.E.

## Author contributions

L. K. and H. v. A. conceptualization; L. K., A. J. d. G., G. M. J., P. A. v. V., and H. v. A. data curation; L. K. software; L. K., G. M. J., P. A. v. V., and H. v. A. formal analysis; L. K., A. J. d. G., G. M. J., X. C., J. C., P. A. v. V., and H. v. A. validation; L. K., A. J. d. G., G. M. J., X. C., K. V., F. M., J. C., P. A. v. V., and H. v. A. investigation; L. K., A. J. d. G., G. M. J., X. C., J. C., and H. v. A. visualization; L. K., A. J. d. G., X. C., J. C., and H. v. A. methodology; L. K. and H. v. A. writing-original draft; L. K. and H. v. A. project administration; L. K. and H. v. A. writing-review and editing; X. C., F. M., and J. C. resources; J. C., P. A. v. V., and H. v. A. supervision; J. C., P. A. v. V., and H. v. A. funding acquisition.

## Supplementary Material

Supporting Information

## Footnotes

3 Please note that the JBC is not responsible for the long-term archiving and maintenance of this site or any other third party hosted site.

## Footnotes

2 The abbreviations used are:

PTM: post-translation modification
HAT: histone acetyltransferase
HDAC: histone deacetylase
qPCR: quantitative PCR.
