# Supplementary material for: Gcn5 and Esa1 function as histone crotonyltransferases to regulate crotonylation-dependent transcription
Source: J Biol Chem. 2019 Nov 7;294(52):20122–34. doi: 10.1074/jbc.RA119.010302 (PMC6937567; doi:10.1074/jbc.RA119.010302)
Supplement: Supporting Information [file supp_294_52_20122__index.html]

Gcn5 and Esa1 function as histone crotonyltransferases to regulate crotonylation-dependent transcription — Gcn5 and Esa1 function as histone crotonyltransferases — Gcn5 and Esa1 function as histone crotonyltransferases to regulate crotonylation-dependent transcription — Gcn5 and Esa1 function as histone crotonyltransferases — Supporting Information 

# Gcn5 and Esa1 function as histone crotonyltransferases to regulate crotonylation-dependent transcription

## Supporting Information

- Supporting Information (to be published online) - S1 Table
- Supporting Information (to be published online) - S2 Table
- Supporting Information (to be published online) - Supporting Information
