# Supplementary material for: Gcn5 and Esa1 function as histone crotonyltransferases to regulate crotonylation-dependent transcription
Source: J Biol Chem. 2019 Nov 7;294(52):20122–34. doi: 10.1074/jbc.RA119.010302 (PMC6937567; doi:10.1074/jbc.RA119.010302)
Supplement: Supporting Information [file supp_RA119.010302_154650_1_supp_424324_q02d62.pdf]

Supporting Information

Gcn5 and Esa1 function as histone crotonyltransferases to regulate crotonylation-dependent transcription

Leonie Kollenstart[1], Anton J.L. de Groot[1], George M.C. Janssen[2], Xue Cheng[3], Kees Vreeken[1], Fabrizio Martino[4], Jacques Côté[3], Peter A. van Veelen[2] and Haico van Attikum[1,*]

This supporting information file contains:

- S1-S11 Figures
- Title to S1 Table
- Title to S2 Table

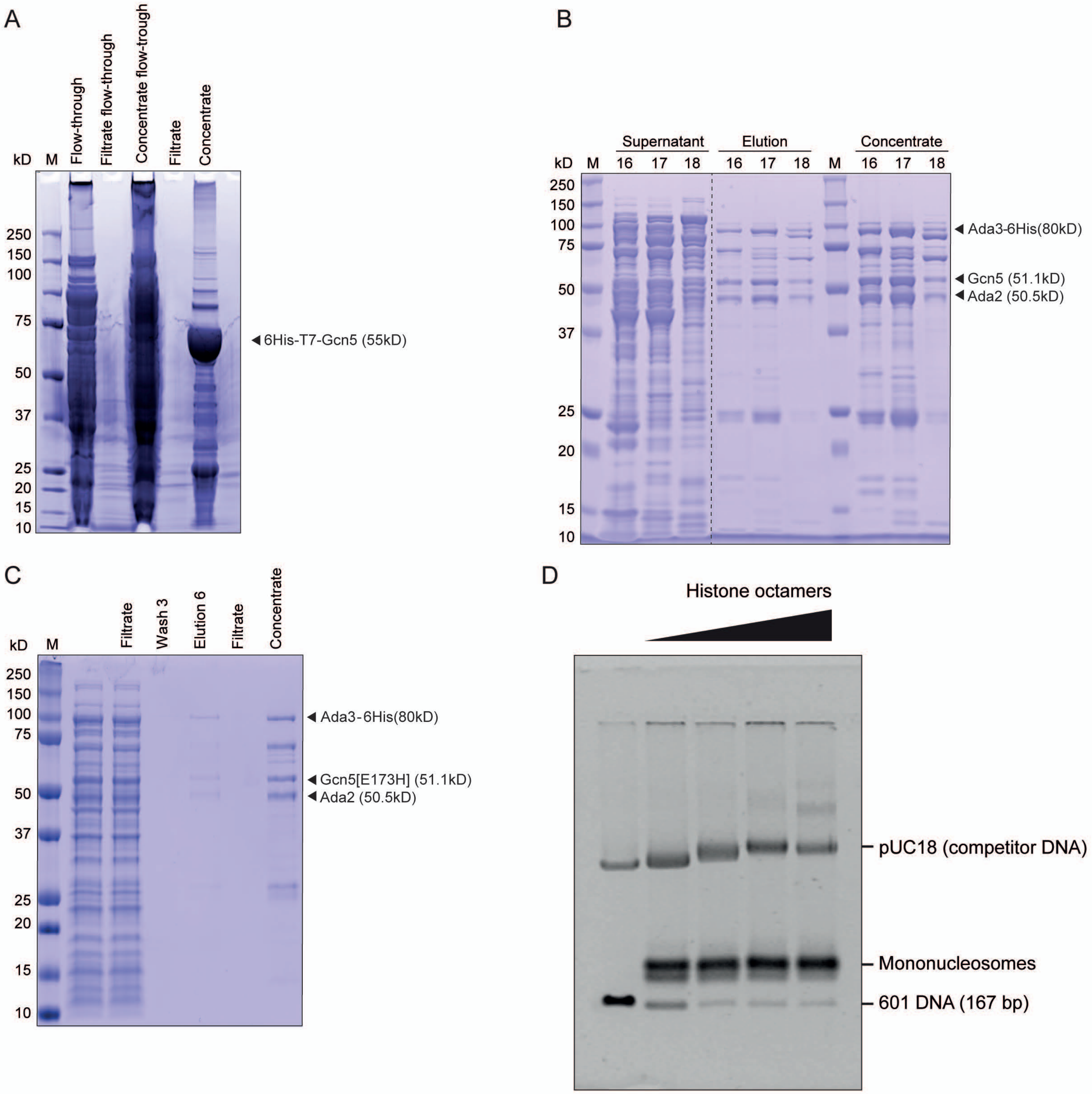

S1 Figure. Purification of recombinant ADA complex and Gcn5, and reconstitution of mononucleosomes.

Coomassie-stained gels of (A) recombinant wild-type ADA complex and (B) catalytic-dead (Gcn5-E173H) ADA complex as purified from *E. coli* following expressed from a polycistronic vector. Fractions 16, 17, 18 were used for purification of the ADA complex. Gels separated by the dotted line originate from the same gel. Coomassie-stained gel of (C) recombinant wild-type Gcn5 as purified from *E. coli* following expressed from a bacterial expression vector (D) Agarose gel of reconstituted mono-nucleosomes containing purified histone octamers, 601 DNA sequence and pUC18 competitor DNA.

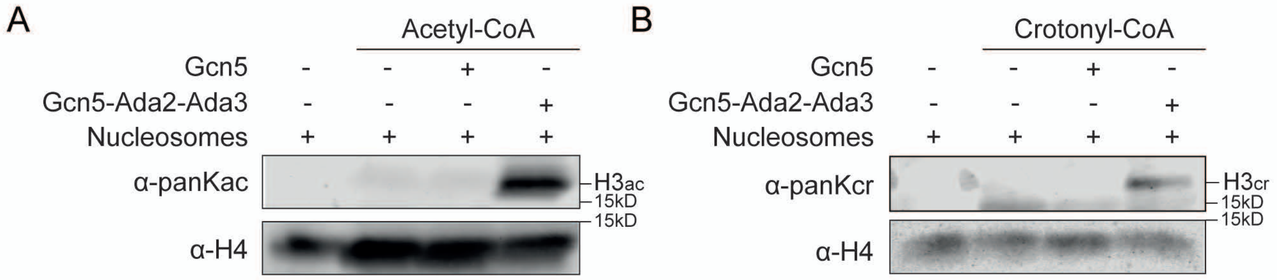

S2 Figure. The ADA complex, but not Gcn5 alone, crotonylates nucleosomes *in vitro*.
Western blot analysis of *in vitro* (A) histone acetyltransferase and (B) histone crotonyltransferase reactions with recombinant wild-type Gcn5 and recombinant wild-type ADA complex and mononucleosomes as a substrate.

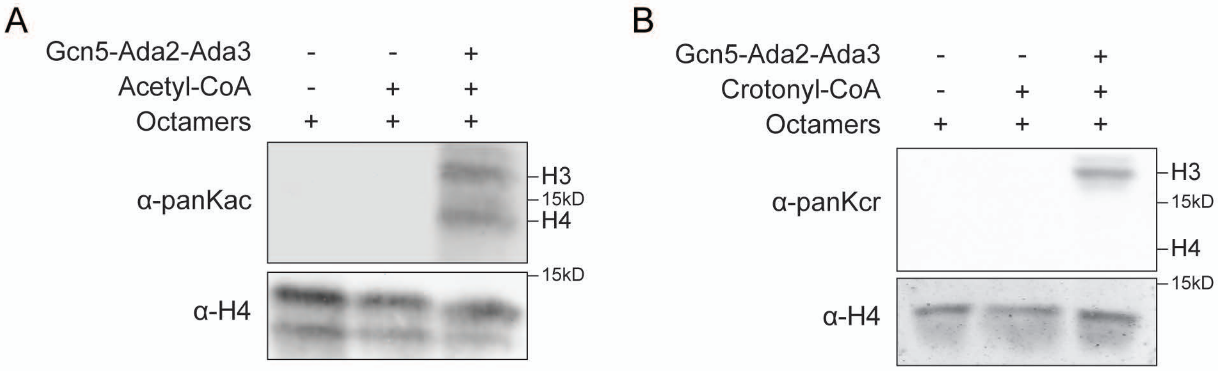

S3 Figure. The ADA complex crotonylates histone octamers *in vitro.*
Western blot analysis of *in vitro* (A) histone acetyltransferase and (B) histone crotonyltransferase reactions with recombinant wild-type ADA complex and histone octamers as a substrate.

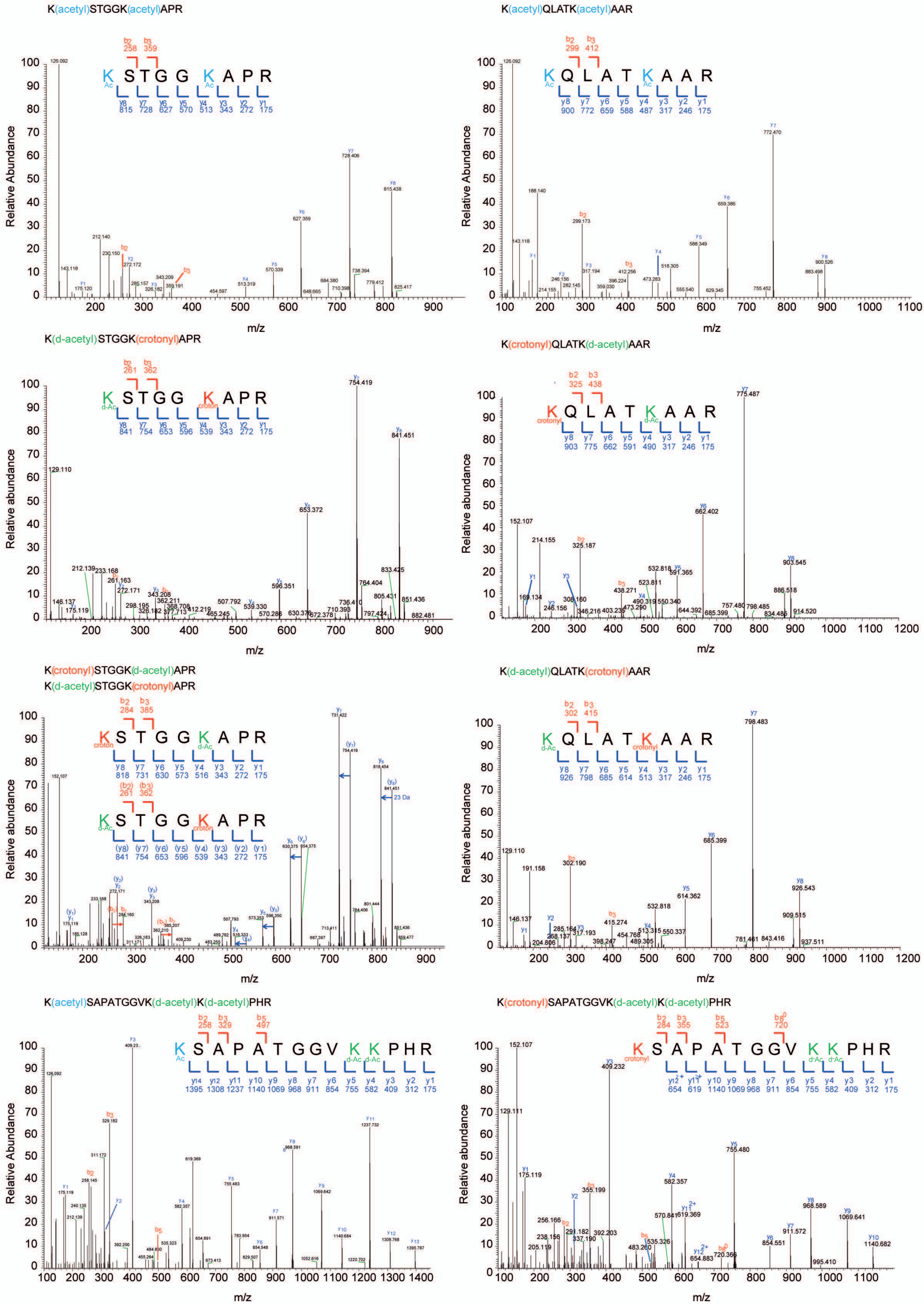

S4 Figure. MS2 spectra peptides acetylated and crotonylated *in vitro* by the ADA complex.

S4 Figure. MS2 spectra peptides acetylated and crotonylated *in vitro* by the ADA complex. Acetylation and crotonylation by the Gcn5-Ada2-Ada3 (ADA) complex is shown in blue and red, respectively while deuteroacetylation (d) is shown in green.

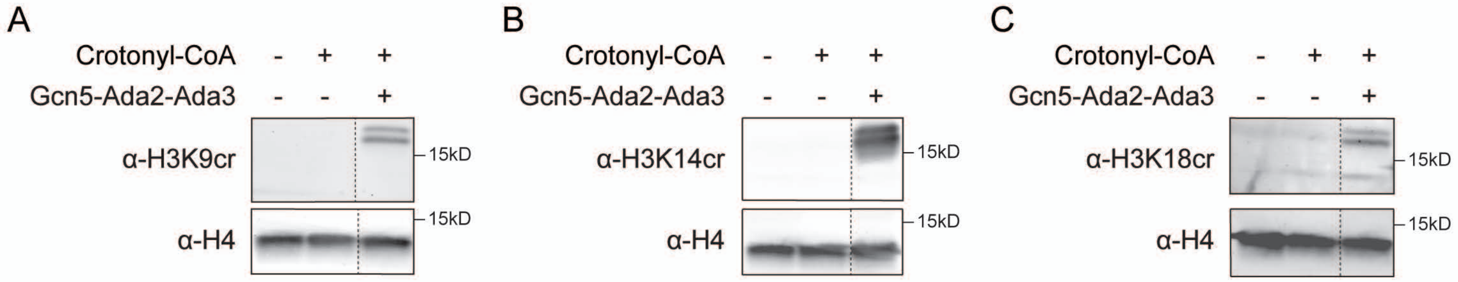

S5 Figure. The ADA complex induces site-specific crotonylation marks on histone H3. Western blot analysis of in vitro histone crotonyltransferase reactions with recombinant wild-type ADA complex using (A) H3K9cr-, (B) H3K14cr- and (C) H3K18cr-specific antibodies. Dotted line separates samples from the same blot.

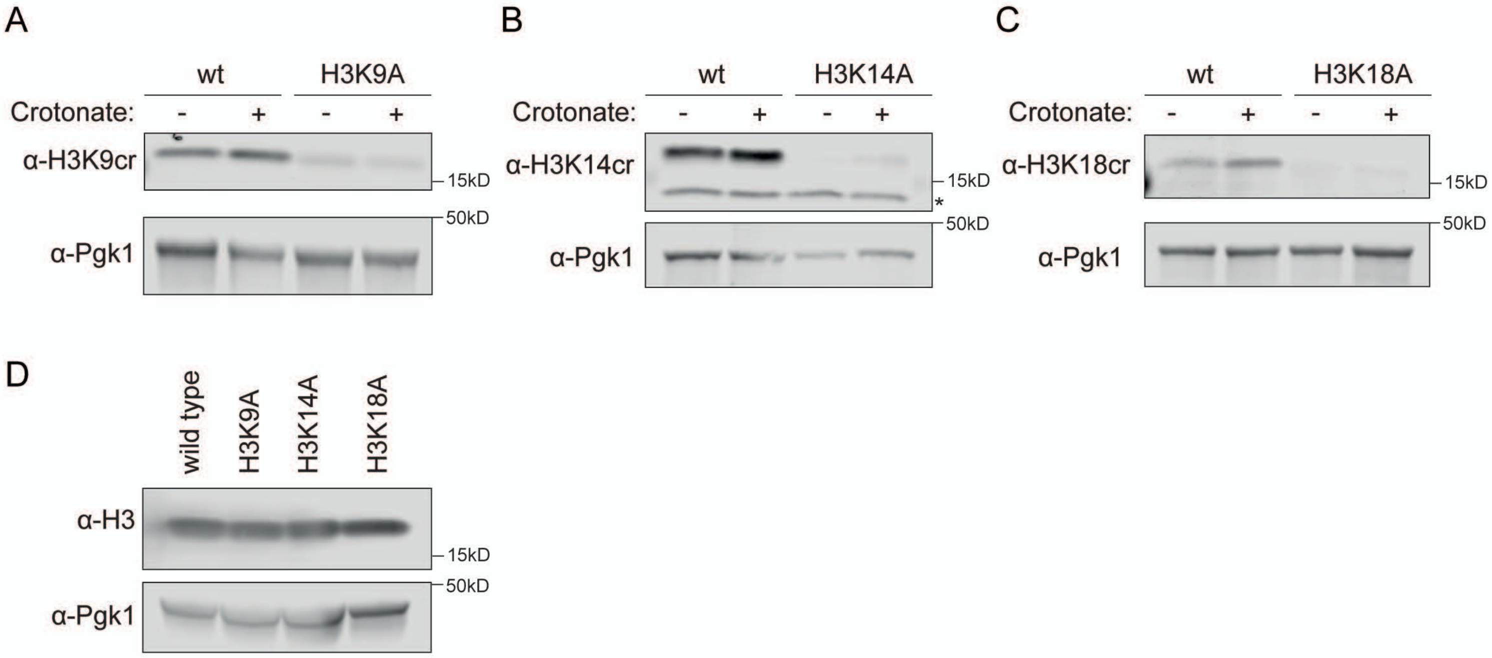

S6 Figure. Site-specific crotonylation antibodies recognize crotonylation marks in yeast chromatin.

Western blot analysis of (A) H3K9cr, (B) H3K14cr, (C) H3K18cr and (D) H3 in whole cell extracts from wild-type yeast and the indicate histone substitution mutants. (A-C) Wild-type and mutant yeast were treated with sodium crotonate for 3.5 hours in the presence of 0.8M sorbitol. Asterisk highlights background bands.

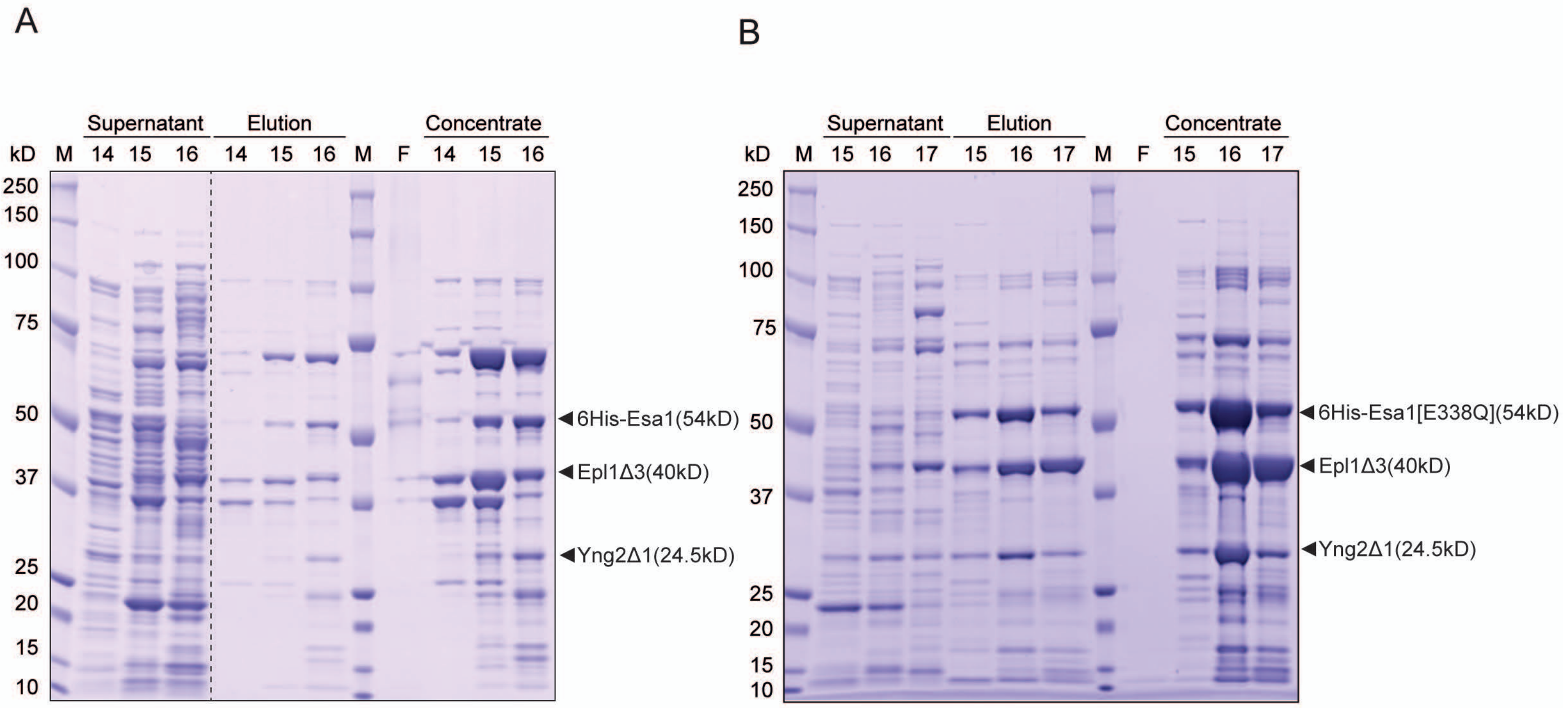

S7 Figure. Purification of recombinant Piccolo NuA4 complexes.

Coomassie-stained gels of (A) recombinant wild-type Piccolo NuA4 complex and (B) catalytic-dead (Esa1-E338Q) Piccolo complex as purified from E. coli following expressed from a polycistronic vector. Epl1 and Yng2 contain deletions to increase the solubility of the proteins. Fractions 16, 17, 18 were used for purification of the wild-type complex and fractions 15, 16, 17 were used for the catalytic-dead complex. Gels separated by the dotted line originate from the same gel.

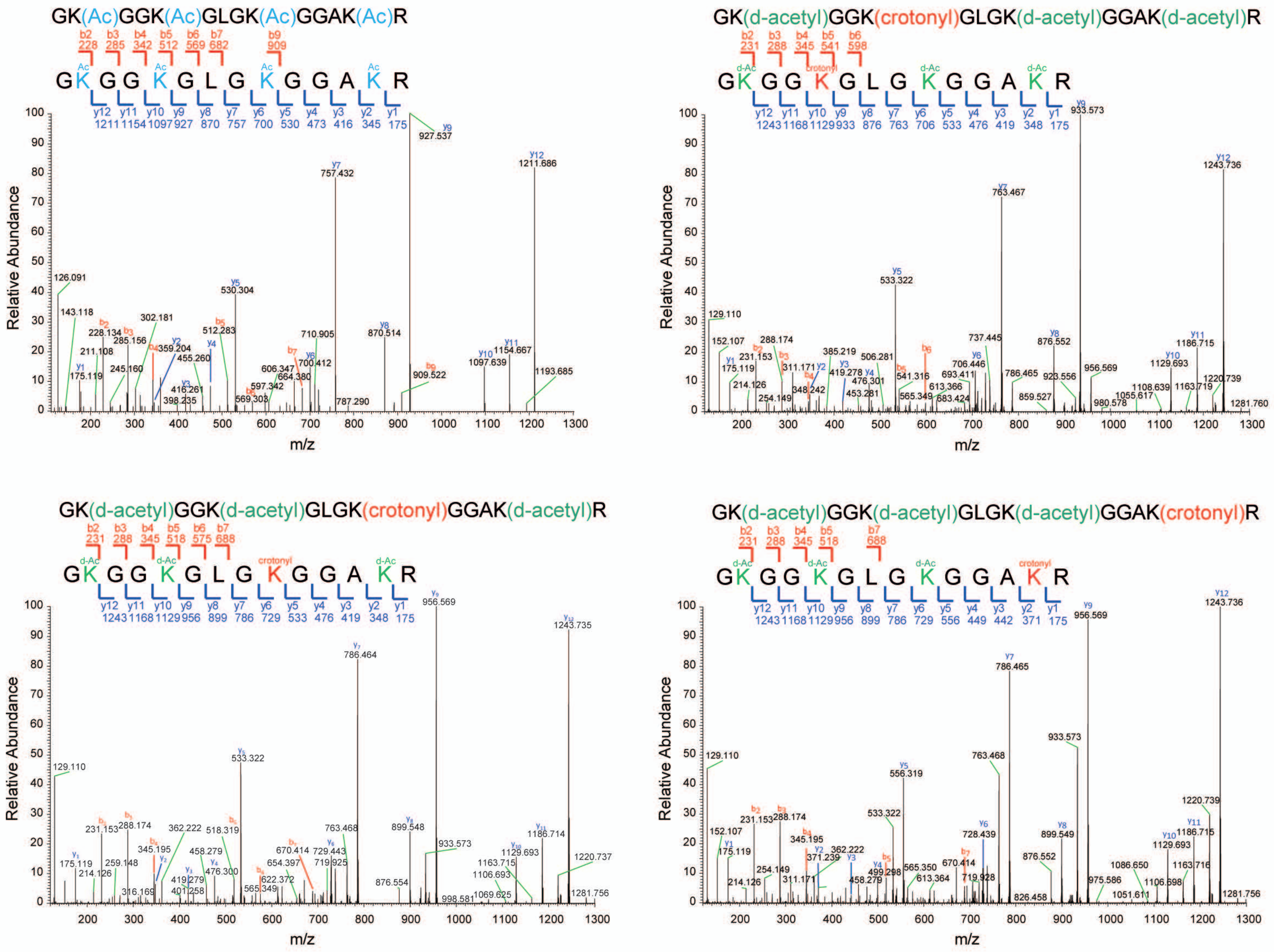

S8 Figure. MS2 spectra of H4 peptides acetylated and crotonylate*d in vitro by* the Piccolo NuA4 complex.

Acetylation and crotonylation by the Esa1-Yng2-Epl1 (Piccolo NuA4) complex is shown in blue and red, respectively while deuteroacetylation (d) is shown in green.

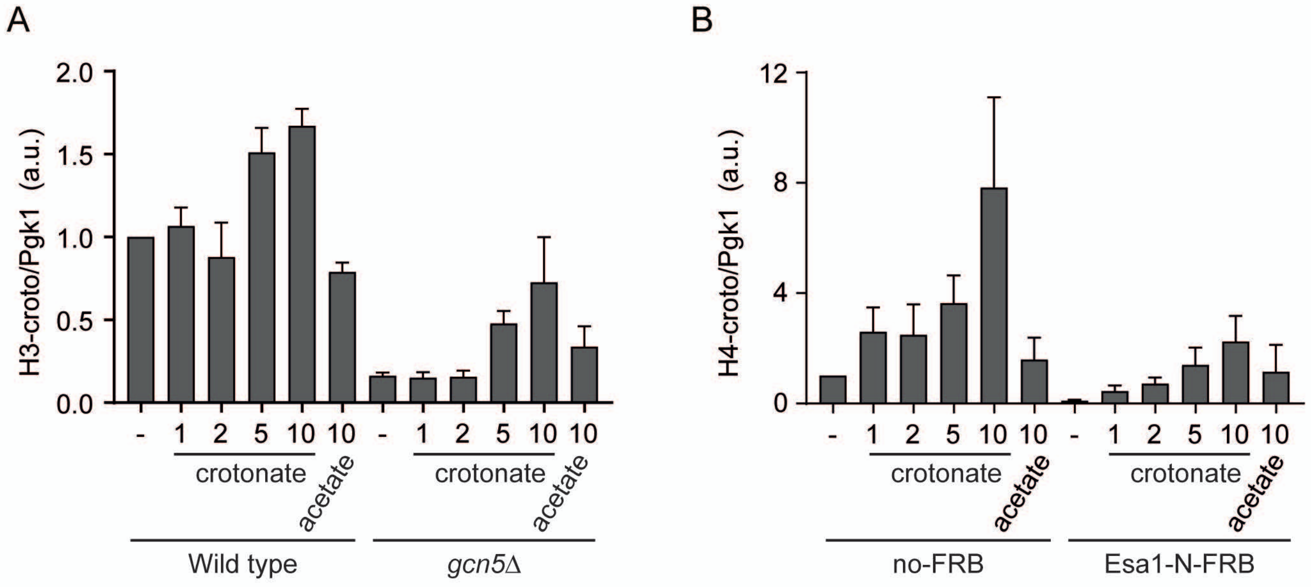

S9 Figure. Quantification of crotonate-induced crotonylation signals.
Quantification of three independent blots of (A) H3-crotonylation signal over Pgk1 in the presence and absence of Gcn5, (B) H4-crotonylation signal over Pgk1 in the presence and absence of Esa1.

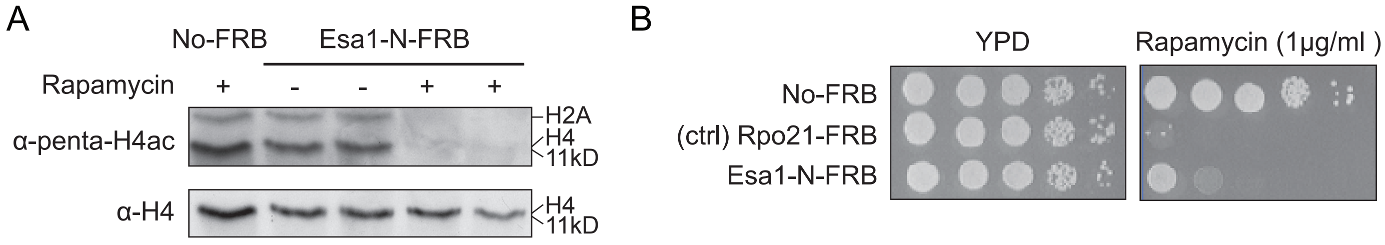

S10 Figure. Validation of the FRB-tagged Esa1 strain.

(A) Western blot analysis of chromatin extracts from cells grown for 3hr with or without the presence of 1μg/ml rapamycin. (B) Spot dilution assay of indicated strains on YPD with and without rapamycin. Rpo21, an essential gene like Esa1, is included as a control.

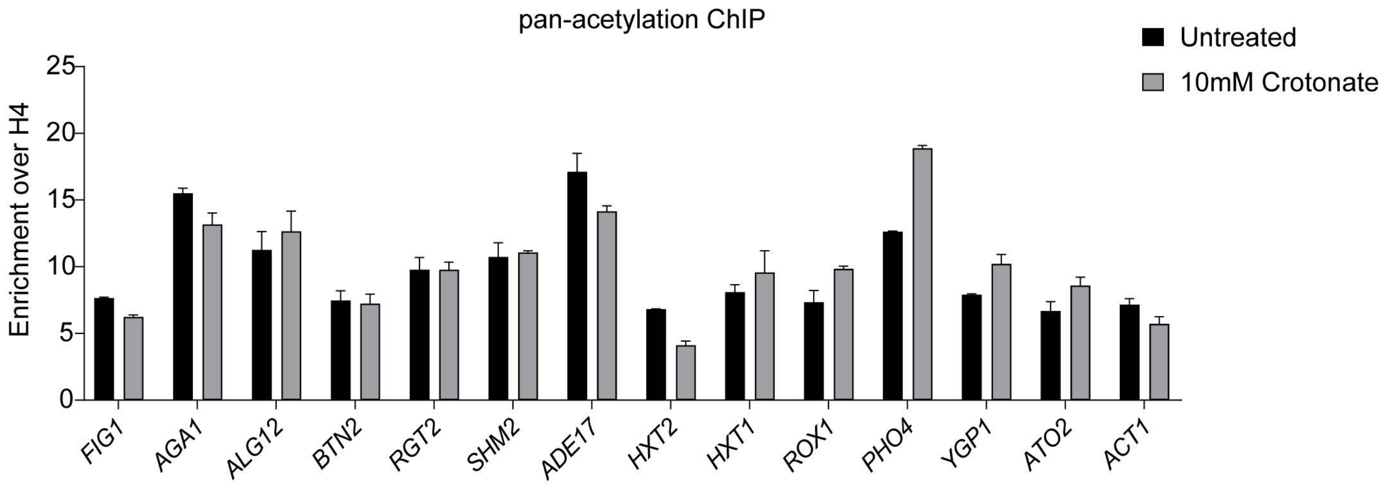

S11 Figure. Crotonate does not induce histone acetylation on gene promoters.
ChIP analysis of acetylation changes on gene promoters after treatment with sodium crotonate for 3.5 hours. Data represent the mean fold enrichment over an IgG control ChIP from two independent experiments ± s.e.m.

S1 Table. **RNA-seq analysis of untreated and crotonate-treated wild-type yeast cells (data source for Fig 4).**

S2 Table. **List of peptides identified in mass spectrometry.**
